# Lutein, Zeaxanthin, and* meso*-Zeaxanthin in the Clinical Management of Eye Disease

**DOI:** 10.1155/2015/865179

**Published:** 2015-12-24

**Authors:** Nicole K. Scripsema, Dan-Ning Hu, Richard B. Rosen

**Affiliations:** ^1^Department of Ophthalmology, The New York Eye and Ear Infirmary of Mount Sinai, New York, NY 10003, USA; ^2^Department of Pathology, The New York Eye and Ear Infirmary of Mount Sinai, New York, NY 10003, USA; ^3^Icahn School of Medicine at Mount Sinai, New York, NY 10029, USA

## Abstract

Lutein, zeaxanthin, and* meso*-zeaxanthin are xanthophyll carotenoids found within the retina and throughout the visual system. The retina is one of the most metabolically active tissues in the body. The highest concentration of xanthophylls is found within the retina, and this selective presence has generated many theories regarding their role in supporting retinal function. Subsequently, the effect of xanthophylls in the prevention and treatment of various eye diseases has been examined through epidemiological studies, animal studies, and clinical trials. This paper attempts to review the epidemiological studies and clinical trials investigating the effects of xanthophylls on the incidence and progression of various eye diseases. Observational studies have reported that increased dietary intake and higher serum levels of lutein and zeaxanthin are associated with lower risk of age-related macular degeneration (AMD), especially late AMD. Randomized, placebo-controlled clinical trials have demonstrated that xanthophyll supplementation increases macular pigment levels, improves visual function, and decreases the risk of progression to late AMD, especially neovascular AMD. Current publications on the preventive and therapeutic effects of lutein and zeaxanthin on cataracts, diabetic retinopathy, and retinopathy of prematurity have reported encouraging results.

## 1. Introduction

Macular pigments are xanthophyll carotenoids that provide the macula lutea with its yellow appearance. Lutein (L), zeaxanthin (Z), and* meso*-zeaxanthin (MZ) are the three major xanthophylls found in the eye. L and Z cannot be synthesized* de novo* and must be acquired from the diet. MZ is a metabolite of L but also can be absorbed from the diet [1]. The highest dietary concentration of L and Z are found in green leafy vegetables, egg yolk, corn, citrus, and other fruits [2]. With the exception of the cornea, vitreous, and sclera, these xanthophylls are found throughout the visual system [3]. The highest concentration of L and Z is in the retina [4]. Macular pigments account for 20–30% of total carotenoids in the human serum, but 80–90% of carotenoids in the human retina [5]. The concentration of L, Z, and MZ in the macula is much higher than concentrations in the serum and liver. This suggests a specific uptake and storage mechanism for L, Z, and MZ in the retina and emphasizes their essential role in retinal function [6]. The aim of this review is to briefly describe the role of these xanthophylls in maintaining visual function. In addition, it provides an overview of current clinical investigations studying the role of macular pigments on visual function and preventing the development and progression of age-related macular degeneration (AMD), retinopathy of prematurity (ROP), diabetic retinopathy (DR), and cataract.

## 2. Lutein, Zeaxanthin, and *meso* -Zeaxanthin in the Retina

Macular pigments have a unique distribution within the retina. Concentrations of L, Z, and MZ are highest in the macula, especially in the center of the macula (the fovea). While zeaxanthin has a peak concentration in the central fovea, lutein predominates in the periphery [7, 8]. The ratio of L to Z in the fovea is approximately 1 : 2.4. Moving eccentrically from the fovea to the periphery, zeaxanthin concentrations decline rapidly while lutein levels slowly rise. Therefore, in the periphery the ratio of L to Z reverses, exceeding 2 : 1 [9]. Bone et al. demonstrated that in the fovea zeaxanthin coexists with its isomer MZ [7]. They proposed that L, MZ, and Z are actually found in equal quantities in the central macula (in an area with 3 mm diameter of the macula). MZ, unlike L and Z, was previously thought to be undetectable in the human liver or serum. Therefore, it was theorized that MZ was a specific metabolite of lutein found only in the retina [10]. The 3 : 1 ratio of L to Z in serum and the 2 : 1 ratio in the fovea support the theory of the conversion of L to MZ in the macula. However, more recently MZ has been detected in the serum, and supplementation trials have demonstrated a significant increase in macular pigments levels after oral supplement with MZ, suggesting that MZ can be absorbed after oral administration and transported to the macula [11]. Supplementation trials involving L, MZ, and Z suggest that MZ may be absorbed and converted in the retina, as supplementation with high dosages of MZ (10 mg MZ, 10 mg L, and 2 mg Z or 17 mg MZ, 3 mg L, and 2 mg Z) resulted in higher macular pigment levels and higher MZ serum levels than supplementing without MZ (20 mg L and 0.86 mg Z) [12]. The results of this trial will be discussed in greater detail below.

Macular pigments are found in their highest concentration in the outer plexiform layer and inner plexiform layer [13]. L and Z have a peak absorbance near 460 nm. In the inner retina they serve as a filter for high energy, short wavelength blue light [13]. This protects the outer retina from photochemical injury easily induced by these high energy wavelengths [14]. They also enhance visual performance by decreasing chromatic aberration and enhancing contrast sensitivity [15–17].

Blue light filtration is one of the many functions of macular pigment [4]. L and Z are also found in the rod and presumably cone outer segments. In the outer retina, macular pigments serve as antioxidants. Photoreceptor outer segments contain chromophores that act as photosensitizers susceptible to oxidative damage. Macular pigments are capable of quenching reactive oxygen species produced from chromophore irradiation, which protects the retina from the deleterious effects of lipid peroxidation [9, 18]. Polyunsaturated fatty acids, especially docosahexaenoic acid (DHA), have high concentrations in the rod outer segments [19]. DHA is highly susceptible to lipid peroxidation and a subsequent cascade of cellular damage. L can return singlet oxygen to the ground state and remove resultant energy as heat, preventing lipid peroxidation. Lutein autoregenerates in the process and is not consumed [20]. This makes L a more efficient quencher of singlet oxygen than other antioxidants such as alpha tocopherol (vitamin E) [21]. Macular pigments are very effective antioxidants, capable of quenching singlet oxygen and triplet state photosensitizers, inhibiting peroxidation of membrane phospholipids, scavenging reactive oxygen species, and reducing lipofuscin formation [22–28].

Although L and Z differ only by the placement of a single double bond, this small alteration in configuration has a great impact on the function of these two carotenoids [29]. Compared to L, Z is a much more effective antioxidant [30]. MZ also has a greater capability of quenching oxygen radicals than L [10]. The functional differences of these carotenoids correlate with the spatial distribution of L, MZ, and Z. The ratio of L to Z varies linearly with the ratio of rods to cones in the fovea. MZ and Z predominate where cone density is highest and risk of oxidative damage is greatest [30, 31]. The macular pigments also differ in other aspects. For example, L has a greater filtering efficacy, and Z is superior in preventing lipid peroxidation induced by UV light [32, 33].

These essential functions of macular pigment decrease oxidative stress in the retina and enhance vision in both normal and diseased retinas.

## 3. Lutein and Zeaxanthin and Visual Function

Macular pigments enhance visual function in a variety of ways. The filtration of blue light reduces chromatic aberration which can enhance visual acuity and contrast sensitivity. L and Z also reduce discomfort associated with glare and improve visual acuity, photostress recovery time, macular function, and neural processing speed.

Discomfort glare is a term used to describe photophobia and discomfort experienced when intense light enters the eye. When testing photosensitivity, subjects are more sensitive to shorter wavelengths of light, which are capable of inducing retinal damage with less energy compared to other wavelengths. Despite increased sensitivity to shorter wavelengths, Stringham et al. found a minimum sensitivity was observed at macular pigment peak absorbance (460 nm). They proposed that photosensitivity serves as a protective function to prevent damage to the eye, and macular pigments could attenuate this visual discomfort by absorbing the high energy wavelengths before they reach the photoreceptor layer [34, 35]. In analyzing the photophobic response produced by glare, subjects with higher macular pigment levels tolerated light better [35]. They also noted that a small increase in macular pigment provided significant improvement in photophobia thresholds and lessened visual discomfort. Similarly, Wenzel et al. also showed a direct correlation between macular pigment levels and photophobia thresholds [36]. This evidence suggests that macular pigment supplementation has a role in reducing discomfort associated with glare.

Disability glare is a term used to describe decreased visual acuity resulting from scattered light, another phenomenon that results from bright light settings. Stringham and Hammond Jr. demonstrated that subjects with higher macular pigment levels maintained acuity better than subjects with lower levels when exposed to both bright white light and short wavelength (blue) light [37]. The response was more exaggerated with the white light, suggesting that macular pigment has a filtering effect integrated across all wavelengths and can reduce disability glare under broad illumination [38, 39]. When patients were supplemented with L and Z, glare disability was improved [40].

Photostress recovery is another parameter of visual performance affected by macular pigments. Photostress recovery is a term used to describe the time necessary to recover vision following exposure to a bright light source. Physiologically, this describes the time necessary for photopigments bleached by a bright light source to regenerate. Stringham and Hammond Jr. demonstrated that subjects with higher macular pigment levels had shorter photostress recovery time when tested with intense short wavelength and bright white light sources [37]. They proposed that macular pigment reduces photostress recovery time by reducing photoreceptor exposure to short wavelength light in the foveal and parafoveal regions. Recovery time for the subject with the lowest macular pigment levels was twice as long as subjects with the highest macular pigment levels [38]. After supplementing patients with L and Z, photostress recovery time was significantly decreased [40]. Hammond et al. reported that daily supplementation with L (10 mg/d) and Z (2 mg/d) for 3 months resulted in significant increase in serum levels of L and Z and MPOD and improvements in chromatic contrast and recovery from photostress in 57 young and healthy subjects as compared with 58 controls [41]. Correlation of MPOD and visual performance also has been studied in patients suffering from eye diseases [17, 42–47], which will be described later.

Nolan et al. performed a randomized, placebo-controlled clinical trial supplementing young, healthy subjects with lutein for 12 months. Their goal was to identify if visual performance could be improved with supplementation in a population with relatively high macular pigment levels considered to be at peak visual performance. They were not able to show a significant change in visual performance in supplemented patients despite doubling serum L levels and significantly increasing central macular pigment levels. They did demonstrate, however, that there was a significant difference in visual performance in subjects in the lowest versus the highest tertile groups [48]. These findings suggest subjects with a sufficient baseline macular pigment level and good visual performance may not benefit from supplementation.

In addition to enhancing visual performance, macular pigments have also been implicated in benefiting neurophysiological health by affecting the complex relationships between optical, neurological, and physiological mechanisms underlying vision. Higher macular pigment levels are attributed to better critical flicker fusion frequency [49], transparency of the crystalline lens [50–52], higher concentrations in the visual cortex [53], and improvements in ERG [54, 55].

Macular pigments are present throughout the visual system, including the brain [53, 56, 57]. Animal models have shown macular pigment optical density (MPOD), a method for quantitating macular pigment levels* in vivo*, is a good proxy for the quantity of xanthophylls in the brain [58]. MPOD correlates with processing speed and cognitive performance in healthy elderly subjects as well as those with mild cognitive impairment [10, 59–61]. Bovier et al. found moderate but statistically significant improvements in both MPOD and cognitive function when supplementing young, healthy individuals considered to be at peak cognitive efficiency [62]. These studies suggest that both young, healthy adults and the elderly population can gain cognitive benefits from L and Z supplementation. Proposed mechanisms for improvement are based in cellular connectivity, as carotenoids may influence the production of connexin proteins that improve intracellular communication [63–65]. This data suggests that patients suffering from poor visual or cognitive performance may experience an improvement in symptoms with increased dietary intake or supplementation of L and Z.

## 4. Age-Related Macular Degeneration

Age-related macular degeneration (AMD) is the most common cause of irreversible blindness in people over the age of 50 in the developed world [66]. Although the pathogenesis of AMD is poorly understood, oxidative stress has been implicated as a major contributing factor. As L and Z are powerful antioxidants selectively absorbed and maintained in the retina, their role in AMD has been studied extensively.

### 4.1. Observational Studies (Dietary Intake of L and Z)

Initial studies focused on the relationship between dietary intake of L and Z and the risk for AMD. While the results of these studies were variable, most suggested that high dietary intake of L and Z is associated with a decreased risk of AMD.

Ma et al. published a systematic review and meta-analysis on this subject [67]. They analyzed six longitudinal cohort studies [68–72] and found that early and late AMD have different relationship with the intake of L and Z. In the late AMD, the pooled relative risk (RR) was 0.74 with 95% confidence intervals (CI) at 0.57–0.97, which indicated that increase in the intake of L/Z was significantly associated with a 26% risk reduction for late AMD. Furthermore, a significant inverse association was observed between L/Z intake and neovascular AMD risk (RR 0.68; 95% CI 0.51–0.92), but not with geographic atrophy. The meta-analysis found that dietary intake of L/Z was not significantly associated with a reduced risk of early AMD.

In addition to the six papers analyzed by Ma et al., there were several other important observational studies published. The Eye Disease Case-Control Study reported that subjects with the highest quintile of carotenoid intake had a 43% reduced risk of AMD compared with subjects in the lowest quintile [73]. Vitamin A, vitamin C, or vitamin E consumption did not provide a similar risk reduction. Similarly, the Blue Mountain Eye Study reported a 65% reduced risk of neovascular AMD between subjects with the highest and lowest intake of L/Z. Subjects above the median carotenoid intake also had a reduced risk of indistinct soft or reticular drusen [69].

In the Age-Related Eye Disease Study (AREDS) Report 12, the relationship between dietary intake of L/Z and late AMD was studied in 4,519 AMD patients. Dietary L/Z intake was inversely associated with neovascular AMD (odds ratio (OR), 0.65; 95% CI: 0.45–0.93), geographic atrophy (OR, 0.45; 95% CI: 0.24–0.86), and large or extensive intermediate drusen (OR, 0.73; 95% CI, 0.56–0.96), comparing the highest versus lowest quintiles of intake, after adjustment for total energy intake and nonnutrient-based covariates. Other nutrients (*β*-carotene, vitamin C, vitamin E, lycopene, etc.) were not independently related to AMD [74].

Furthermore, participants from the Rotterdam Study were enrolled into a case-control study investigating whether dietary nutrients could reduce the genetic risk of early AMD. A total of 2,167 participants from the population-based Rotterdam Study at risk of AMD were followed up for a mean of 8.6 years. They reported that high dietary intake of nutrients with antioxidant properties such as L and Z, *β*-carotene, omega-3 fatty acids, and zinc reduced the risk of early AMD in those at high genetic risk [75]. This is the first report to evaluate both genetic and environmental risk factors for AMD.

### 4.2. Observational Studies (Serum Levels of L/Z)

Several studies evaluated the serum levels of L/Z. The Beaver Dam Eye Study found that L/Z serum levels did not correlate with AMD [76]. Gale et al. examined the relationship between AMD and plasma L/Z levels in 380 AMD patients and found that the risk of AMD (early or late) was significantly higher in individuals with lower plasma Z levels. Subjects with the lowest third Z levels had double the risk of AMD compared to those with the highest third. Risk of AMD was also associated with plasma L levels; however, the relationship between L and AMD was not significant [77].

### 4.3. Studies on* In Vivo* Macular Pigment Levels

A case-control study of human donor eyes by Bone et al. demonstrated that donors with AMD had significantly lower levels of macular pigment (MP) compared to eyes without; and donors with the highest quartile of L/Z had an 82% lower risk of having AMD compared to donors in the lowest quartile [78]. This was the first study to report decreased retinal levels of MP in patients with AMD, which correlated with previous studies analyzing serum carotenoid levels. The authors did note that decreased MP could at least in part be attributable to the disease process [79].

Subsequently concentrations of L and Z in the retina have been studied extensively. Macular pigment levels within the retina are easily measured* in vivo* as macular pigment optical density (MPOD) with heterochromatic flicker photometry and retinal reflectometry [80, 81]. MPOD correlates with dietary intake of carotenoid-rich foods [52] and circulating serum L and Z levels [82]. MPOD in healthy subjects shows an age-related decline, and healthy eyes at risk for AMD have significantly lower MPOD than healthy eyes not at risk [83]. The CAREDS study, a prospective cohort analysis of nearly two thousand postmenopausal women, did not find a correlation between MPOD and AMD [84]. However, other studies have reported a correlation of lower MPOD in eyes with AMD, and several supplementation trials studying subjects with AMD reported a decreasing MPOD in their placebo group over the course of the trial [42, 85, 86]. Lower levels of macular pigment have also been associated with other risk factors for the disease, including a positive family history of AMD, tobacco use, and obesity [87].

### 4.4. Xanthophyll Supplementation Trials

After the established correlation between the risk of AMD and low serum and retinal concentrations of L and Z, supplementation trials were initiated. These trials have shown extremely consistent results as compared to any other single nutrient supplementation trial.

The first supplementation trial reported was the Veterans Lutein Antioxidant Supplementation Trial (LAST). This was a double-masked, placebo-controlled trial that investigated lutein supplementation alone compared to combined supplementation (lutein, other carotenoids, antioxidants, vitamins, and minerals) in 90 patients with dry AMD and geographic atrophy. Both groups demonstrated a significantly increased level of MP, improved visual acuity (VA) at near, and improved contrast sensitivity (CS). The disease progression was halted with supplementation over the course of the 12-month study. While the duration of the study was short and study group numbers were small, few studies have monitored the effects of MP supplementation alone compared to combined supplementation [17].

The Age-Related Eye Disease Study (AREDS) was one of the largest and earliest supplementation trials which demonstrated that subjects with extensive intermediate-sized drusen, at least one large druse, noncentral geographic atrophy, or advanced AMD in one eye had 25% reduced risk of severe vision loss at 5 years if supplemented with vitamin C (500 mg), vitamin E (400 IU), b-carotene (15 mg) with or without zinc (80 mg), and copper (2 mg cupric oxide) [88]. The treatment effect appeared to persist following 5 additional years of follow-up after the trial ended [89]. However, the effects of L and Z were not evaluated in this study.

Weigert et al. evaluated the role of lutein supplementation in MPOD, visual acuity, and macular function (assessed with microperimetry) in intermediate to advanced AMD. A total of 126 patients were randomized to L (20 mg daily for 3 months and then 10 mg daily for 3 months) or placebo for a period of 6 months. Supplementation significantly increased MPOD. There was a trend toward increased macular function and visual acuity that was not statistically significant [47].

Ma et al. evaluated the role of macular pigment supplementation in early AMD over 48 weeks. A total of 107 subjects were randomized to a placebo, L (10 mg/day), L (20 mg/day), or L (10 mg/day) and Z (10 mg/day). They reported a significant increase in MPOD in all study groups with the exception of the 10 mg lutein group. There was no change in the placebo group. Subjects with the lowest baseline MPOD had the greatest increase in MPOD regardless of supplementation. Visual acuity (VA) improved in all treatment groups, but not significantly. Contrast sensitivity (CS) was significantly different at 48 weeks in all treatment groups. The authors noted that MPOD was significantly increased at 24 weeks, while VA and CS did not show improvement until 48 weeks, suggesting that visual function cannot be improved until MPOD levels reach and maintain high levels [44].

The CARMIS study reported a significant improvement in CS and NEI visual function questionnaire at 12 and 24 months in AMD patients supplemented with vitamin C (180 mg), vitamin E (30 mg), zinc (22.5 mg), copper (1 mg), L (10 mg), Z (1 mg), and astaxanthin (4 mg) compared to controls. VA was not significantly improved until 24 months [46], consistent with other supplementation trials.

The LUTEGA study evaluated the long term effects of L, Z, and omega-3 fatty acid supplementation on MPOD in 145 dry AMD patients randomized to placebo, daily or twice daily dosage of supplement. The supplement provided was L (10 mg), Z (1 mg), and omega-3 fatty acid (100 mg DHA, 30 mg EPA). After 12 months, MPOD increased significantly in supplementation groups and decreased significantly in controls. VA also improved compared to placebo. There was no significant difference in accumulation of MPOD between the two dosage groups. No progression was noted in any of the participants [43].

The CLEAR study evaluated the effects of L (10 mg) supplementation on early AMD subjects over a 12-month period. This group reported a significant increase in mean MPOD after 8 months of supplementation, with no change in the control group. VA improved in the study group and declined slightly in the placebo group. There was also an increase in serum L levels in the study group, increasing anywhere from 1.8 to 7.6 times the baseline values. Those with lower baseline serum levels tended to have greater improvements, but the response to supplementation varied markedly between individuals [45].

The CARMA study investigated the role of L and Z with other antioxidant vitamins and minerals in subjects determined to be at highest risk of progression to advanced AMD. A total of 433 subjects were randomized to the placebo or supplementation group. Patients were supplemented with Ocuvite twice daily (L 12 mg, Z 0.6 mg, vitamin E 15 mg, vitamin C 150 mg, zinc oxide 20 mg, and copper gluconate 0.4 mg). VA improved after 12 months of supplementation but was not significant until 24 months. CS was also improved, but not significantly. Fewer eyes in the active group progressed compared to controls (41.7% versus 47.4%, resp.). Macular pigment values in the study group demonstrated a small increase over time, while the placebo group steadily declined. Serum concentrations of all antioxidants were increased after six months of supplementation. The increases in these serum levels did not correlate with improvements in VA. However, an increase in serum L levels was associated with slower progression of AMD. A similar pattern was seen with serum Z levels but did not achieve statistical significance [42].

Liu et al. performed a meta-analysis which compared the results of the above-mentioned seven randomized, double-blind, placebo-controlled trials, including the LAST, Weigert et al., Ma et al., CARMIS, LUTEGA, CLEAR, and CARMA studies [17, 42–47]. Four of the seven studies demonstrated an increase in VA with supplementation. A stronger effect was noted for studies using higher doses of supplements. The analysis demonstrated that supplementation is associated with significant improvements in VA and CS in a dose-response relationship. A linear association of MPOD and an increase in VA and CS was also noted. Compared with early AMD patients, late AMD patients tended to have a less significant improvement in VA. This was attributed to the loss of macular photoreceptors in the late stage of the disease [90].

After the release of several smaller supplementation trials mentioned above, the Age-Related Eye Disease 2 Study (AREDS2) was published. AREDS2 was a multicenter, randomized, double-masked, placebo-controlled clinical trial following 4,203 participants with intermediate AMD or large drusen in 1 eye and advanced AMD in the fellow eye for approximately 5 years. Participants were assigned to one of four groups: placebo, L (10 mg) and Z (2 mg), omega-3 fatty acids (DHA 350 mg and EPA 650 mg), or a combination of L, Z, and omega-3 fatty acids. In addition they were given either the original AREDS formulation or some modification of the original formulation (eliminating *β*-carotene, lowering zinc dose, or a combination of the two). The original analysis id not find significant effects from xanthophyll supplementation. However, a secondary analysis (2014) of the effects of L/Z on AMD progression in AREDS2 revealed definitively positive results [91]. The authors reanalyzed the results of AREDS2 by analyzing L/Z versus no L/Z and comparing L/Z and *β*-carotene. In the analysis of L/Z versus no L/Z, the development to the late AMD was significantly decreased in patients treated with L/Z; the risk ratio (RR) of late AMD was 0.90 (95% CI, 0.82–0.99; *P* = 0.04). Analyses of the comparison of L/Z versus *β*-carotene also showed significant decrease of risk of development of late AMD and neovascular AMD in L/Z group but did not appear to influence development of geographic atrophy. In analyses restricted to eyes with bilateral large drusen at baseline, the comparison of L/Z versus *β*-carotene showed even better effects, RR of 0.76 for progression to late AMD, and RR of 0.65 for neovascular AMD. The totality of evidence regarding beneficial and adverse effects of *β*-carotene in AREDS2 and other studies suggests that L/Z is more appropriate than *β*-carotene for the new AREDS2 formulation.

These studies established that structural changes in the retina can be achieved with supplementation, and over time supplementation appears to affect visual acuity. More recent studies have evaluated functional changes in carotenoid supplementation with the multifocal electroretinogram (MfERG). As a secondary analysis to their initial study, Ma et al. compared 107 subjects with early AMD randomly assigned to one of four treatment groups (placebo, L 10 mg/day, L 20 mg/day, or L 10 mg/day and Z 10 mg/day) comparing MfERG responses at baseline, 24, and 48 weeks. They demonstrated that early functional abnormalities in the central retina of subjects with early AMD at baseline could be improved with supplementation of L and Z. They attributed these improvements to the significant increase in MPOD seen at both 24 and 48 weeks [55]. Berrow et al. reported a similar study with smaller sample size randomizing 14 subjects with AMD to placebo or supplementation with Ocuvite Duo for 40 weeks (L 12 mg, Z 0.6 mg, omega-3 fatty acids consisting of EPA 240 mg and DHA 840 mg, vitamin E 15 mg, vitamin C 150 mg, zinc oxide 20 mg, and copper gluconate 0.4 mg). MfERG was performed at 20, 40, and 60 weeks (20 weeks after supplement withdrawal). There was no significant difference in MfERG results during the course of the trial. However, subjects in the treatment group had significant improvement in MfERG results compared to baseline that regressed at the final visit 20 weeks after the supplement was removed [54].

These trials suggest that with long term supplementation of antioxidants in patients with AMD increase in macular pigment in the retina allows for improved macular function, visual acuity, and contrast sensitivity. Evidence suggests with supplementation serum levels increase quickly, macular pigment increases over a period of several months, and a minimum of one to two years is necessary before improvements in visual function reach statistical significance. Recent studies show that macular pigment levels continue to increase with long term supplementation [12, 92]. Subjects with lower baseline macular pigment levels often show the greatest response to supplementation. The supplementation of L and Z also can retard the progress of intermediate AMD to late AMD, especially in regard to neovascular AMD.

Previous studies mainly investigated the preventive and therapeutic effects of L and Z; very little was known on the effects of MZ on the AMD. Recent reports investigating the ratio of L, Z, and MZ supplementation suggest supplementing with a higher proportion of MZ leads to higher MPOD values and an improvement in CS [12, 92] indicating that including MZ in a supplement may confer benefits for the treatment of early AMD.

While the last two decades of research have provided many insights into the role of macular pigments and other antioxidants in AMD, future research studies investigating the optimal antioxidant supplement, the role of early supplementation, the relationship of MPOD as a risk factor for disease onset and progression, and the impact of genetic risk factors are necessary to better understand the disease process and provide more therapeutic options to patients with AMD.

## 5. Other Retinopathies

The role of carotenoids in age-related macular degeneration has been studied extensively. The encouraging results have led to subsequent investigations into the role of antioxidants in other diseases, including diabetic retinopathy and retinopathy of prematurity. The retinal ischemia in these conditions can lead to neovascularization, hemorrhage, and blindness. Oxidative stress plays a role in the pathogenesis of both conditions, and early evidence suggests antioxidant supplementation may prevent disease progression [93].

### 5.1. Retinopathy of Prematurity

In retinopathy of prematurity, premature infants are exposed to higher oxygen tensions compared to conditions* in utero*, which downregulates VEGF generation and the development of normal retinal vasculature. The relatively avascular retina then becomes hypoxic with increasing metabolic demand, which initiates expression of proangiogenic factors. This stimulates aberrant angiogenesis, leading to intravitreal neovascularization [94–97]. The ischemic retina in ROP also has an imbalance between the generation and sequestration of reactive oxygen species (ROS). The developing retina in premature infants is particularly susceptible to oxidative damage for several reasons. The high proportion of long chain polyunsaturated fatty acids (PUFA) [98, 99] leaves the retina susceptible to lipid peroxidation which can damage retinal tissues. In addition, preterm infants have reduced levels of antioxidants compared to full term infants, as they are often produced or accumulated later in gestation [100]. Hence, in preterm infants the endogenous antioxidant system is overwhelmed, leading to a prooxidative state capable of causing irreversible damage to various cell structures. Biomarkers of retinal stress, such as lipofuscin, show rapid increase in RPE cells during the first few years of life. This suggests even infants without ROP are at risk [101, 102]. Antioxidants can protect retinal cells from oxidative damage and have inhibited microvascular degeneration in animal models of diabetic retinopathy and oxygen-induced retinopathy [103, 104]. The relative deficit of antioxidants in preterm infants and the growing evidence from animal studies suggest a possible role for antioxidant supplementation in the prevention of ROP progression.

During fetal development L is the dominant retinal carotenoid [10]. Z and MZ slowly accumulate with time. The presence of L in umbilical cords at birth indicates there is placental transfer to the fetus, with concentrations peaking in the third trimester [105]. A randomized controlled trial of 150 newborns demonstrated that neonatal supplementation of L in the first hours of life increased biological antioxidant potential and reduced levels of total hydroperoxide [106]. Subsequently, four randomized controlled trials investigated the relationship between xanthophylls and ROP [107–110]. L was the primary xanthophyll used in the supplementation trials due to its predominance in the infant retina.

Two multicenter placebo-controlled randomized clinical trials studying ROP prevention supplemented preterm infants (<33 weeks of gestational age) with 0.5 mL daily dosage of 0.14 mg L and 0.0006 mg Z via oral feeds of maternal milk, donor human milk, or preterm formula [107, 108]. The supplemented groups showed reduced incidence of ROP compared to control groups (6.2% versus 10.3% and 19% versus 27%, resp.). In addition, while not statistically significant, supplemented subjects with ROP showed a 50% decrease progression from early to threshold and higher ROP stages compared to controls.

A third clinical trial investigated the effect of weight-based dosages, as AMD trials have suggested better outcomes with higher carotenoid doses. This trial did not show a difference in ROP incidence with weight-based doses, but the study was limited by small sample size [109].

The fourth multicenter randomized controlled trial compared carotenoid levels in preterm infants fed formula with and without L, lycopene, and *β*-carotene to carotenoid levels in full term infants fed human milk. A secondary outcome was visual complication. ROP incidence was similar between the premature formula fed groups, but the supplemented group had less progression to severe ROP versus the control group (8% versus 28%). The supplemented group also had similar plasma L levels compared to full term infants fed human milk. The study also compared L levels with photoreceptor activity and found that normal plasma lutein levels at 50 weeks of age correlated with a saturated response amplitude in rod photoreceptors and rod photoreceptor sensitivity [110]. The authors suggest that L may play a role in photoreceptor maturation and visual acuity in the developing retina.

To date no clinical trials have specifically tested the hypothesis that L affects ROP outcomes. While future supplementation trials monitoring long term outcomes in ROP would be beneficial, current evidence suggests a role for carotenoid supplementation in the prevention of ROP and normal photoreceptor development in preterm infants.

### 5.2. Diabetic Retinopathy

In diabetic retinopathy, prolonged hyperglycemia causes oxidative stress via several different pathways [111–116]. Evidence from animal models suggests L and Z can block the pathways leading to oxidative stress by quenching oxygen radicals and therefore preserving retinal function [117–121]. Animal studies have found that the neuroprotective activities of L prevent neuronal loss in the diabetic retina [120, 121].

While a number of studies have examined the role of carotenoids in the development of diabetes mellitus (DM), there are a limited number of studies examining their role in the development of diabetic retinopathy. A serum analysis of patients with Type II DM demonstrated that patients with a higher concentration of serum L, Z, and lycopene compared to serum alpha-carotene, *β*-carotene, and *β*-cryptoxanthin had a 66% reduction in the risk of diabetic retinopathy after adjusting for confounding variables [122]. Studies of MPOD have shown subjects with Type II DM have lower MPOD compared to age-matched normals. In comparing the diabetic subjects, those with retinopathy had lower MPOD than subjects without, and MPOD levels correlated with glycosylated hemoglobin levels [123]. While there are not currently any supplementation trials that evaluate the role of L and Z in the prevention or treatment of DR, one study demonstrated that daily supplementation of nonproliferative diabetic subjects with 6 mg L and 0.5 mg Z increased MPOD, improved VA and CS, and increased foveal thickness compared to controls [124].

Evidence supporting the role of macular pigments in the prevention and treatment of retinopathies is currently limited, but animal models and early human supplementation trials suggest there is a role for lutein and zeaxanthin in reducing oxidative damage and possibly preventing disease progression.

## 6. Cataracts

Age-related cataracts are another leading cause of blindness in the United States and worldwide. Treatments that can delay the progression of lens opacities have been studied extensively as this would reduce the burden of disease and reduce healthcare costs. Numerous studies have investigated the role of dietary nutrients in the development of cataracts or need for cataract surgery [125–130]. Specifically, antioxidants are of interest for their potential role in reducing oxidative damage leading to cataract formation. L and Z are the only carotenoids found within the human lens, although in significantly lower concentrations compared to the retina [131]. Approximately 74% of L and Z are located in the epithelium and cortex, where the lens is exposed to oxygen in the surrounding aqueous humor [132]. Proposed functions include preventing oxidative stress and lipid peroxidation in the epithelial cells.

The first trial to suggest a relationship between vitamins and minerals and cataractogenesis was a trial in Linxian, China, aimed at reducing the risk of esophageal and gastric cancer in a nutritionally deprived population. The initial trial compared multivitamin/mineral supplement and placebo, and the second trial compared 4 different supplements (retinol/zinc, riboflavin/niacin, ascorbic acid/molybdenum, and selenium/vitamin e/*β*-carotene). The authors found the risk of nuclear cataract progression over 5 to 6 years was decreased by at least 36% when supplementing with multivitamins [133]. However, the AREDS clinical trial found no effect of nutrients supplementation on the development of lens opacity. There was an equal proportion of subjects that underwent cataract surgery in treatment and control groups [134]. Similar results were reported for the Physicians' Health Study and the Women's Health Study. None of these studies have investigated the effects of L and Z.

While the trials mentioned above were underway, Hammond et al. demonstrated that higher levels of MPOD correlated with a more transparent lens. They hypothesized that higher concentrations of xanthophylls in the retina correlate with higher concentrations in the lens, impacting the rate of cataract progression [52]. Two early epidemiologic studies support these findings. Both demonstrated subjects with the highest quintile of L and Z had a 20% reduced risk of developing cataract compared to subjects in the lowest quintile [51, 135]. The Beaver Dam Eye Study reported similar results regarding L intake and nuclear cataract. They found that increased L intake at baseline decreased the risk of nuclear opacities among subjects younger than 65 by 50% compared to those with the lowest L intake. There was no significant influence in older subjects [136].

A retrospective study by Gale et al. demonstrated a 50% reduced rate of posterior subcapsular cataract in subjects with higher plasma L concentrations. High plasma vitamin C, vitamin E, and Z were not associated with a decreased risk [137]. Berendschot el al. examined serum antioxidant levels and MPOD and found an association between higher MPOD and a lower incidence and progression of cataracts [138]. Vu et al. studied 3,271 subjects in Australia and reported a 36% reduced rate of nuclear cataract in those with the top quintile of lutein and zeaxanthin intake combined. There was no correlation with cortical or posterior subcapsular cataracts [139].

Another population-based study (Pathologies Oculaires Liees a l'Age (POLA) study) investigating plasma L and Z levels of 899 subjects found those with the highest quintile of plasma Z had a significantly reduced risk of AMD, nuclear cataract, or any cataract [140]. There was no association between serum L or serum L and Z combined. While the numerous observational studies provide varied results on the impact of carotenoid supplementation on nuclear and posterior subcapsular cataracts, the general trend suggests there is a role for L and Z in prevention of cataract progression.

In a ten-year prospective study examining serum carotenoid levels in 35,551 female subjects, Christen et al. demonstrated that women in the highest quintile of L and Z intake had an 18% lower risk of developing cataract compared to those in the lowest quintile [141].

Subsequently a few prospective supplementation trials have investigated the role of carotenoids in the prevention of cataract formation. Omedilla et al. studied the visual effects of L supplementation on subjects with cataracts in a double-blind placebo-controlled study. Visual acuity and glare sensitivity were improved after 2 years of supplementation with L 15 mg. However, sample sizes of the treatment and study groups were small (*n* = 5 and *n* = 6, resp.). They did not evaluate cataract progression [142]. AREDS2 evaluated cataract formation as a secondary outcome and is currently the largest clinical trial investigating carotenoid supplementation and cataract progression. They reported that L and Z supplementation had no statistically significant overall effect on rates of cataract surgery or vision loss related to cataract progression. While the epidemiologic studies provide encouraging data, there are a limited number of randomized controlled trials to support the role of L and Z supplementation in the prevention of cataractogenesis.

## 7. Conclusions

Three xanthophylls (L, Z, and MZ) are found selectively within retina, concentrated in the macula, and have been appropriately referred to as macular pigments. Epidemiological studies have revealed that low macular pigment levels are associated with higher risk of AMD. Several large observational studies demonstrated that high dietary intake and higher serum levels of L and Z are associated with a lower risk of AMD, especially late AMD. Randomized controlled clinical trials have revealed that supplementation of L and Z increases macular pigment density, improves visual function, and decreases the risk of progression of intermediate AMD to late AMD, especially neovascular AMD. Future studies may include additional assessments of the relationship between macular pigment and different genotypic and phenotypic forms of AMD, the optimum dosages of L, MZ, and Z, and the possible synergistic effects associated with supplementing with other nutrients. Current studies on preventive and therapeutic effects of L and Z on ROP, DR, and cataract have yielded varied results. Further investigations are necessary to fully understand the role of macular pigment in the prevention and treatment of eye diseases such as AMD, ROP, DR, and cataract.
